# Exploring Potential COPD Immunosuppression Pathways Causing Increased Susceptibility for MAC Infections among COPD Patients

**DOI:** 10.3390/clinpract11030077

**Published:** 2021-09-09

**Authors:** Shafaa Munjal, Shalok Munjal, Jingya Gao, Vishwanath Venketaraman

**Affiliations:** 1 Palm Desert High School, Palm Desert, CA 92260, USA; shafaa.munjal@myds.us; 2 Department of Psychiatry, State University of New York, Buffalo, NY 14215, USA; shalokmu@buffalo.edu; 3 College of Osteopathic Medicine of the Pacific, Western University of Health Sciences, Pomona, CA 91766, USA; jingya.gao@westernu.edu

**Keywords:** non-tuberculous mycobacterial pulmonary disease, chronic obstructive pulmonary disease, *M. avium* infections, tuberculosis, *Mycobacterium avium* complex

## Abstract

Although there has been a drastic decline in the cases of Tuberculosis in the United States, the prevalence of infections caused by *Mycobacterium avium* Complex (MAC) has steadily increased in the past decades. *Mycobacterium avium* (*M. avium*) is one of the most abundant microorganisms in the MAC species. The mycobacterium genus is divided into two major groups: tuberculosis causing mycobacteria and non-tuberculous mycobacteria. MAC is most prominent among the non-tuberculous mycobacteria. MAC is an opportunistic pathogen that is present in soil, water, and droplets in the air. MAC infections can result in respiratory disease and can disseminate in affected patients. MAC infections are especially prevalent in patients with preexisting respiratory conditions such as Chronic Obstructive Pulmonary Disease (COPD). COPD is one of the most common lung conditions in the world with the primary cause being smoking in developed countries. COPD involves chronic inflammation of lung tissue resulting in increased susceptibility to infection. There is a lack of research regarding the pathophysiology that leads COPD patients to be susceptible to MAC infection. Our review paper therefore aims to investigate how the pathogenicity of MAC bacteria and immune decline seen in COPD patients leads to a greater susceptibility to MAC infection among COPD patients.

## 1. Introduction

Chronic Obstructive Pulmonary Disease (COPD) is a chronic inflammatory condition of the pulmonary system that results in obstructive airflow from the lung tissue. Patients with COPD exhibit symptoms of breathing strain, mucus filled sputum, wheezing, chest tightness, fatigue, and increased cough. COPD incidence is directly linked to chronic exposure to pulmonary irritants such as pollution, particulate matter, and most commonly smoking. COPD is broadly classified into Emphysema and Chronic Bronchitis. Emphysema is a condition where the lung alveoli suffer extensive damage, while Chronic Bronchitis is a long-term inflammation of bronchioles and bronchi that transport air to and from the alveoli. Currently, around 6.6% of the US population or 16.4 million US individuals suffer from COPD. Patients with a history of lung disease such as COPD have an increased risk of *Mycobacterium avium* Complex (MAC) colonization [1,2]. In patients with COPD, the risk of contracting a mycobacterial infection is 15 times higher than that of the general population.

Mycobacterium genus was first discovered on 24 March 1882 by Dr. Robert Koch. In the 100 years that followed, other researchers studied MAC and its virulence factors. The most famous species of this genus is Mycobacterium Tuberculosis. However, overall, the mycobacterium genus has more than a hundred different species under its umbrella that have been discovered so far. Mycobacterium genus can generally be differentiated into two main classes: tuberculoid-mycobacterium versus *Mycobacterium avium* Complex (MAC) [2] Mycobacterium genus is unique in the way that its cell membrane contains mycolic acid. Most of the mycobacterium are aerobic organisms, which do not produce any spores, are nonmotile, and are generally curvilinear to linear shaped [3]. The fact that their cell envelope is composed of lipid-rich material renders them acid-fast. Additionally, mycobacterial peptidoglycan contains lipids in place of proteins and polysaccharides. These properties make the genus neither gram-positive or gram-negative. The cell membrane of mycobacterium is quite similar to cell membranes of other bacteria, but it still differs from them with respect to the presence of lipoarabinomannan (LAM), lipomannan, and phosphatidylinositol mannosides [3].

The objective of this review is to synthesize the current literature on *Mycobacterium avium* Complex (MAC) susceptibility among COPD patients and discuss some possible physiological and metabolic pathways that result in this increased susceptibility among COPD patients. Additionally, this paper intends to bring light to some key considerations in the treatment of patients affected.

## 2. Materials and Methods

The focus of this review is to explore the connection between *M. avium* and COPD. The majority of the articles discussed the high incidence of *M. avium* infections in patients with COPD. The search strategy started with searching for *M. avium* and COPD in abstracts, and expanded to pathogenesis of mycobacterium, and decreased immune response of individuals with COPD and *M. avium* separately. The search criteria included keywords such as: *M. avium*, COPD, immunodeficiency, and immunology. M. tb and tuberculosis were excluded in other searches to narrow down MAC as a topic of discussion. The search engine utilized was PubMed, with the majority of articles sourced from the same. Some of those articles included translations of literature in other languages. Specific case studies relating to *M. avium* and COPD were sought out and discussed.

## 3. Results/Discussion

### 3.1. Pathogenesis of Mycobacterium avium Complex (MAC)

*Mycobacterium avium* Complex (MAC) includes three major species: *M. avium*, *M. intracellulare*, and *M. chimaera* [4]. These three species are difficult to distinguish and cause disease with similar symptomatology. These diseases generally have a chronic indolent course and are quite resistant to antibiotics. In immunocompromised individuals, such as patients with HIV, Wiscott-Aldrich syndrome, severe combined immunodeficiency disease (SCID), Primary Immunodeficiency Diseases (PIDD), and cancers of the immune system, e.g., leukemias and lymphomas, mycobacterium can dissipate from its usual pulmonological location to other organ systems, causing disseminated infections [5,6]. Since mycobacterium are facultative intracellular species, they can adapt to thrive both within the human cells and outside them. MAC generally live in natural environments and infect humans through inhalation of environmental particles [3]. After inhalation, MAC has the propensity to infect the mucosal epithelial cells lining the human lung tissues. This activates macrophages to fight MAC and the collection of macrophages form a granuloma to contain and inhibit the growth of MAC bacteria [7]. These granulomas increase in size and form nodules that damage the bronchi, forming Nodular Bronchiectasis, and decrease the mucociliary activity in the lung. Additionally, the nodular bronchiectasis also diminishes the immune response to MAC in the lungs [8]. These processes are slow and gradual in nature.

If an infected patient is already predisposed to MAC due to diseases such as HIV and inherited immunodeficiency gene defects, the MAC can disseminate to the lymphatic system and infect other organ systems [9]. Patients with diseases, such as COPD, Bronchiectasis, and cystic fibrosis are also very susceptible to MAC infections. Some of the symptoms of MAC infection include: extreme fatigue, low grade fever, night sweats, and unexplained loss of appetite and weight, shortness of breath, chest pain, and recurring respiratory infections [10]. If the MAC infection becomes disseminated, the patient will likely have organ specific physical exam finds, such as hepatomegaly and lymphadenopathy, along with abnormal blood lab values such as anemia, pancytopenia, increased transaminase, and increased alkaline phosphatase [5].

### 3.2. Prevalence of Mycobacterium avium Complex (MAC)

Mycobacterial infections are globally increasing despite the decline of tuberculosis [1]. Tuberculosis-causing bacteria include M. tb and Mycobacterium leprae resulting in tuberculosis and leprosy, respectively. MAC, also known as Atypical Mycobacterial infections, are other species in the Mycobacterium genus that do not cause tuberculosis or leprosy. MACs are composed of quite a few species, the most prevalent of them being *M. avium*, *M. intracellulare*, and *M. chimaera*. Infections with these MAC species can result in high variability in symptoms [11]. In the United States the incidence of tuberculosis has drastically dropped since World War 2, with a peak during the 1980s at the beginning of the HIV surge, and a steady decline from 1993 to 2019 according to the CDC [12].

The incidence of MAC infections is increasing continuously worldwide. Studies conducted in Belgium [13], Germany [14,15], South Korea [16], Japan [17], Brazil [18], and more showed the increase in incidence of MAC causing pulmonary disease, specifically MAC in the past 15 years. Most of the studies concluded that the recovery rate and infection rates of MACs were increasing in the past decade, with the highest prevalence in the elderly and individuals with existing respiratory predispositions [14].

### 3.3. MAC Assessment Toola and Clinical Diagnosis of Mycobacterium avium Complex (MAC) Infection

With the rise of MAC infections in the recent decades, some studies propose that the improvement in diagnosing MAC could be a contributing factor in the increased incidence reported. There is high host variability in patients with MAC disease, however individuals with MACs are usually elderly females who are thin and tall. They have also identified the extent to which smoking history, scoliosis, and pectus excavatum can result in MAC infection [5]. In a retrospective study conducted in South Korea by Park et al. [16], pleural effusion was studied with the most common species involved being MAC. The individuals were divided into two groups—MAC causing pleuritis and MAC without pleuritis. The study showed that patients in the MAC causing pleuritis group had less nodular bronchiectatic lung features and a lower treatment success rate than the MAC without pleuritis group. This study demonstrated another aspect of the variability of MAC infections that should be considered in formulating a treatment plan in patients with active MAC infections.

MAC infections are typically diagnosed by sputum cultures and acid-fast staining. These diagnostic tests require time and resources. There are many studies that identify those at risk for MAC infections. The Leicester Cough Questionnaire (LCQ) and the St. George Respiratory Questionnaire (SGRQ) were both studied as ways to assess health related quality of life (HRQ) for patients with MAC [19]. The SGRQ was developed to assess the quality of life for patients with COPD but it is not a dedicated assessment for MAC infections [20]. The LCQ is often used to study coughs in patients. Factors considered included BMI, physical, social, and psychological aspects related to their disease process. After analyzing the correlation between SGRQ and LCQ on MAC patients, it was concluded that LCQ may provide use in the assessment of HRQOL in patients with MAC lung disease.

Clinically, in health care settings, the diagnosis of MAC is done with a combination of microbiology lab results, clinical and radiological criteria [10]. Clinical phenotypes of MAC include: Fibrocavitary disease and Nodular Bronchiectatic disease. Fibrocavitary disease is primarily in the superior area of the lungs, focal or cavitary in nature, and affects both males and females. Nodular Bronchiectatic disease is primarily focused on the middle lung lobes, with centrilobular nodular form, with females having a higher incidence rate than males. Nodular bronchiectatic disease has a higher incidence among smokers, and therefore by inference may be more likely found among COPD patients [21]. In 2020, American Thoracic Society, European Respiratory Society, and other published an official clinical practice guidelines for the treatment of MAC [22]. According to the guidelines, MAC requires the presence of pulmonary and systemic symptoms, nodules/cavities on Chest X-ray or bronchiectasis with multiple small nodules on CT, with greater than 2 positive sputum cultures or 1 positive bronchiolar lavage or 1 positive biopsy [23].

### 3.4. Treatment of Mycobacterium avium Complex (MAC) Infection

The treatment is begun if the patient has a known infectious presentation with lung cavitation, increased inflammatory markers (ESR, CRP), positive lung cultures with more virulent MAC species, any form of immunodeficiency, and severe constitutional symptoms. The first line of treatment could be conservative in nature such as physiotherapy, nutrition, albuterol, ipratropium, prednisone, and others. If the symptoms and hemodynamic signs continue to worsen, then antibiotic treatment can be used. The treatment for Nodular bronchiectatic disease is Azithromycin, Rifampicin, and Ethambutol three times weekly. Cavitary and refractory disease requires the same treatment, but with an addition of intravenous Amikacin; and these antibiotic regimens can vary from three times per week to daily. It is advised to continue the antibiotics regimen for about 12 months after getting the first negative sputum culture. Additionally, after diagnosing patients with active infections, patients are advised to limit exposure to soil and environment since MAC are prevalent there. If MAC disseminated to lymph nodes, some patients might benefit from surgical excision of those infected lymph nodes [22].

### 3.5. Pathogenesis of Chronic Obstructive Pulmonary Disease (COPD)

COPD is a leading factor in increasing the risk of a patient developing a MAC infection. COPD is a progressive inflammatory lung disease that leads to airflow limitation. Patients with a history of lung disease such as COPD have an increased risk of MAC colonization [2]. In patients with COPD, the risk of contracting a mycobacterial infection is 15 times higher than that of the general population [24]. One of the main causes of COPD is smoking. According to American Lung Association, almost 90% of COPD cases were the result of smoking history. Although smoking is a common cause, other risk factors such as second-hand smoking and exposure to air pollution invoke the same result. Exposure to second-hand smoking during childhood can elevate the risk of developing COPD in adulthood [25]. Air pollution also exacerbates the symptoms of COPD. Patients with COPD are especially vulnerable to pollutants and the damaging consequences that they have. Other than exposure to air pollution and first and secondhand-smoking, those with alpha-1 antitrypsin deficiency also develop COPD. In this case, COPD is formed because of a genetic condition that affects how the patient’s body produces alpha-1. The alpha-1 antitrypsin protein is a glycoprotein produced in the liver. It is essential because it protects the lung’s alveoli from damage, while the immune system agents fight infections. The sensitive lung tissue of individuals with alpha-1 deficiencies are destroyed, which contributes to the development of COPD [26].

The most common conditions of COPD are emphysema and chronic bronchitis. The primary symptom of emphysema is shortness of breath. In the lungs of emphysema patients, the alveoli are damaged, which negatively affects the exchange of oxygen and carbon dioxide. This occurs due to the formation of large air pockets in the lungs as a result of alveoli deterioration. The eventual destruction of alveoli causes a reduction in the surface area usable for gas exchange [27]. Other symptoms of emphysema include: fatigue, weight loss, coughing with mucus, and wheezing. Chronic bronchitis includes several symptoms that overlap with emphysema. These include: fatigue, wheezing, and shortness of breath. However, a significant symptom that distinguishes it from emphysema is chronic productive cough. Chronic coughing is most notably associated with chronic bronchitis. Patients with chronic bronchitis have frequent coughing that can also produce excess mucus. It is the product of the airways becoming filled with mucus. This productive cough lasts for at least three months a year for two consecutive years. COPD causes deficiencies in the patient’s immune system that leaves them susceptible to mycobacterial infections.

In COPD patients, the resident lung microbiome is eventually altered. Alteration of the lung microbiome causes pathogen colonization [28]. Respiratory tract opportunistic pathogens such as S. aureus and *p*. aeruginosa are also present in the airway of the COPD patients. The pathogens colonize the respiratory tract and result in the production of biofilms that prevent opsonization by antibodies [29]. The changes that occur in the immune system of a COPD patient increase the likelihood of MAC infections. COPD places patients at a high risk of mycobacteriosis development, especially those with bronchiectasis. In the pathogenesis of COPD, the changes that occur leave immunocompromised individuals susceptible to MAC infections.

### 3.6. Immunosuppressant Effects of Steroids in COPD Patients

Chronic Obstructive Pulmonary disease results in chronic inflammation, which causes a positive feedback loop in worsening COPD. Consequently, to mitigate this process, COPD exacerbations are treated by prescribing corticosteroids which help in the reduction of inflammation. Corticosteroids do this by downregulating inflammatory cytokines, reducing oxidative stress, causing a reduction in mast cells, eosinophils, neutrophils, T-lymphocytes, macrophages, GM-CSF, reducing IL-6 and IL-8 from bronchial epithelial cells, and lowering C-Reactive proteins. This results in shorter hospital stays, reduction in COPD exacerbation, decreased relapse rate, and in improved response of patients to bronchodilators. In cases of extreme COPD exacerbations, the use of corticosteroids may even increase FEV1 (Forced Expiratory Volume in 1 s), a shorter time of ventilators, reduced ICU hospitalizations, and overall reduced hospital stay. If chronic inflammation caused by COPD is left unaddressed, it can lead to dysregulation of the balance between damaging inflammation and curative healing processes. Corticosteroids help reduce the destructive effects of inflammation, helping in a speedy recovery [30].

However, these benefits of corticosteroids in COPD come with significant side effects, particularly with respect to increased susceptibility to opportunistic infections. In a case-control study conducted by Vincent X. Liu, they investigated the relationship between Corticosteroid use and MAC infection [31]. They found that patients using corticosteroids had increased odds of developing MAC infection. Additionally, the duration of corticosteroids was positively correlated with increased MAC infection severity. Glucocorticoids have a multifactorial effect on the patient’s adaptive and innate immune system. Glucocorticoids downregulate the transcription of genetic material that gets translated into pro-inflammatory cytokines and chemokines, cell-adhesion molecules, and other proteins that sustain the initiation and maintenance of inflammation [32]. Some of the pro-inflammatory genes that are downregulated by corticosteroids include NF-κB and AP-1 [33]. Glucocorticoids can also upregulate genes that code for phenylethanolamine *n*-methyltransferase (PNMT) [33], dual specificity phosphatase (DUSP) 1, IκB, IL-10, glucocorticoid-induced leucine zipper (GILZ) [34], and others that further suppress inflammation and diminish the response of monocytes, macrophages, antigen-presenting cells in T cells, neutrophils, and pituitary folliculostellate cells resulting in a significant reduction in the efficacy and number of innate and adaptive immune cells [35]. In addition to switching on and off genes, glucocorticoids also influence chromatin remodeling that are highly precise in terms of conformational changes in the DNA structures of genes that upregulate inflammation and downregulate the healing process. Consequently, there is an up-regulation of cytokines and pro-inflammatory proteins that augment the HPA axis, resulting in elevated plasma cortisol levels and elevated plasma 11-dehydrocorticosterone, acting in a positive feedback-loop to diminish the body’s immune system response. This results in increased susceptibility to opportunistic infections, such as *Mycobacterium avium* Complex (MAC) Mycobacterium [35].

COPD treatment includes long-term use of corticosteroids that can result in the immunosuppression of innate and adaptive immune cells, including CD4 cells, lymphocytes, macrophages, neutrophils, and up-regulation of anti-inflammatory proteins. This impedes the ability of the host to fight infections. MAC leads to a chronic infection that can stay in the lungs forming nodules, biofilms, and live-in granulomas, and can cause chronic lung disease. Patients using corticosteroids are similar to immune-deficient patients, such as HIV + patients, where MAC lung infections can even spread to other organ systems via lymphatic systems, causing a disseminated infection [9]. Consequently, it is no surprise that cross-sectional and case-control studies have shown that COPD patients who chronically use corticosteroids are more prone to MAC lung disease than regular non-chronic steroids using COPD patients [36].

### 3.7. Redox Imbalance in COPD Patients

Lung anatomy exposes the lung directly to environmental toxins. This exposure increases the lung’s oxidative stress. COPD patients generally have high exposure to smoking and other inhaled toxins that result in high oxidative and carbonyl stress in the lungs. In COPD patients, the oxidative burden of inspired environmental toxins exceeds the lung’s inherent anti-oxidative defense. This causes chronic damage to the lung tissue, resulting in production of reactive organic molecules that accelerate the damage of neighboring tissue, thereby, increasing the carbonyl stress. In COPD, the chronic burden of oxidative and carbonyl species causes a positive feedback loop that continues long after COPD patients cut out smoking. In addition to environmental toxins causing lung tissue damage, the acute exacerbations of COPD can itself result in the body’s own innate and adaptive immune cells producing reactive oxygen species that increase the oxidative and carbonyl stress in the lungs. This stress can result in mitochondrial damage, autoimmune disease, activation of pro-inflammatory markers, increased mucus production, increased bronchial constriction, tissue remodeling, and overall worsening of both COPD symptoms and acute COPD exacerbations [37].

### 3.8. Glutathione Deficiency in COPD and MAC Infected Patients

One of the most important anti-oxidative markers of the human body is Glutathione (GSH). Glutathione (γ-glutamylcysteinylglycine, GSH) is an important thiol tripeptide antioxidant that maintains cellular redox state and cellular homeostasis in a variety of cellular processes within mammalian cells. GSH binds to the oxidative species and gets oxidized by Glutathione Peroxidase enzyme, thereby, reducing the body’s oxidative and carbonyl stress. Therefore, it plays a crucial role in securing the lung cells and lung tissue function. However, the excess production of reactive species in COPD patients can inundate the antioxidant ability of GSH present in the lungs, resulting in damage to cellular homeostasis and metabolism of pulmonary tissues [38]. Excess oxidative stress among COPD patients may be another factor resulting in a higher prevalence of MAC mycobacterium in the COPD patient population. Since the oxidative stress results in the pulmonary tissue damage, anatomic changes, unregulated immune response to pathogens, dysregulation of cytokines, and increased inflammatory response in the body, it can be inferred that COPD patients have a lower ability to contain mycobacterium infections, thus, resulting in a much higher susceptibility to succumbing to MAC infections.

### 3.9. Increased Susceptibility for MAC Infections among COPD Patients

In a case study conducted in Poland by Wyrostkiewicz et al., a 63-year-old male with a history of smoking and COPD was studied after worsening symptoms and MAC infection [24]. The patient’s X-rays showed chronic bronchitis and bronchiectasis with thickening in the lower lung lobes. A chest CT was obtained, showing a 5 cm lesion in the right lung with enlarged lymph nodes and fluid in the pleural effusion on the same side. The patient reported increased productive cough and difficulty breathing on exertion with fever and weight loss. He was treated in the past with corticosteroid, cholinolytic, and beta-antagonist medications. The patient’s quantiferon TB was negative with a history of childhood tuberculosis. After a 4-week culture, the patient was diagnosed with *M. avium* infection. The study concluded with the increased susceptibility to MAC infections in patients with COPD [24]. Due to the increased risk of opportunistic infections in patients with COPD and the commonality of MAC, it is important to consider diagnostic testing early to start the proper treatment.

MAC is an opportunistic bacterium that infects immunocompromised individuals such as those with COPD or HIV. In a case study conducted in Switzerland, Bachofner, Ikenberg, Schulthess, and Nemeth discussed the inflammatory syndrome caused by *M. avium* infection in a patient with preexisting HIV infection [6]. Patients with HIV/AIDS and chronic pulmonary disease frequently have pulmonary infections attributed to *M. avium* [39]. The cell block revealed granulomatous inflammation with clustering of epithelioid histiocytes in the lymph node. This type of cell formation is present in chronic inflammations, similar to how it presents in patients with COPD.

### 3.10. Immune Alterations Due to MAC

Interferon-γ (IFN-γ) is a cytokine involved in innate and adaptive immunity and functions to stimulate natural killer cells and macrophages. IFN-γ is also important in the regulation of T lymphocyte reaction with dendritic cells. Deficiency in Interferon-γ Receptor 1 (IFNγR1) is associated with Mendelian Susceptibility to Mycobacterial Disease (MSMD) a genetic inheritance of immunodeficiency that increases the susceptibility of mycobacterial infections. Dotta et al. investigated immunodeficient patients with mycobacterium infections and found that patients undergoing active *M. avium* infections had a lower expression of cytokines and lower count of dendritic cells [40]. Dendritic cell maturation is dependent on T-cell response to IFN-γ and IL-12, a defect on either of the respective receptors can result in dendritic cell deficiency. Dendritic cell deficiency has also been reported in patients with monocytopenia with MAC syndrome. Although COPD is not a genetic inherited disease, there is immune dysfunction in patients with COPD that predispose them to MAC infections [37].

In a prospective study conducted by Shu et al., patients with MAC lung disease were compared to healthy individuals to evaluate the responses of tumor necrosis factor-α (TNF-α) and IFN-γ with MAC [8]. Patients with active *M. avium* infections had decreased TNF-α and IFN-γ activity responses as well as decreased stimulation from IL-12 on peripheral blood mononuclear cells. After the patients were treated for their MAC infections, there were increases to the previously mentioned factors. The study proposed that continual infection of MAC can result in cell immunity suppression. In patients with MAC infections, they discovered higher programmed cell death in lymphocytes. It was also mentioned that CD4+ and CD25 lymphocytes had increased expression of programmed cell death, suggesting the pathway involved in inhibiting immunity [7].

Damaged lung tissue in COPD and associated immunodeficiency can increase the susceptibility of infections of opportunistic pathogens with *M. avium* making up a large percentage due to the ubiquitous presence. Inflammation in the lungs of patients with COPD can expose the fibronectin matrix for *M. avium* to bind to and grow [41]. Through various studies it is also proposed that *M. avium* infections can result in chronic inflammation and reduction of the immune response, leading to increased susceptibility to MAC and other mycobacteria.

### 3.11. Parallels between HIV-Aids Patient’s Systemic Immunosuppression and COPD Induced Localized Immunosuppression

HIV (human immunodeficiency virus) is a virus that attacks the body’s immune system. If HIV is not treated, it can lead to AIDS (acquired immunodeficiency syndrome). HIV primarily attacks the immune system, eventually resulting in a state of immune-system failure, resulting in increased susceptibility in fighting most infectious diseases. HIV preferentially infects CD4 cells in the first few weeks of the infection. CD4 cells play an important role in signaling other immune cells in combating pathogenic microorganisms. Since the HIV virus lowers the number of active CD4 cells, the body is not able to muster up a well-coordinated systemic immune response to the infectious pathogen. Consequently, HIV patients are susceptible to MAC infections since there is substantial systemic immunosuppression among HIV-AIDS patients. A parallel to this systemic immunosuppression can be drawn out in COPD patient’s localized immunosuppression in the patient’s pulmonary tissues. COPD results in systematic destruction of lung tissue, uncoordinated response of cytokines, immunosuppression of innate and adaptive immune cells, and redox imbalance in lungs and glutathione deficiency. Overall, this results in COPD patients exhibiting a localized immunosuppression in lungs and greater susceptibility to MAC infections.

## 4. Conclusions

In this article, the correlation between MAC infections and COPD has been thoroughly evaluated in order to provide a concise overview of the topic. The overall finding is that patients with COPD have increased susceptibility to MAC infections, specifically *M. avium* [42]. In the United States, cases of Tuberculosis have significantly declined, however, MAC infections have risen greatly. MAC infections have become more prevalent globally as well according to studies done in Belgium [14], Germany [15], South Korea [16], Japan [17], and Brazil [18]. COPD is a leading factor in increasing the risk of developing a MAC infection. Illnesses such as COPD leave patients predisposed to lowered or weakened immune responses which contributes to the patients’ susceptibility to MAC colonization. COPD is most commonly caused by smoking, however other causes such as exposure to second-hand smoking, air pollution, and alpha-1 antitrypsin deficiency also invoke the same result [25]. COPD leads to chronic inflammation of the lungs along with alterations in the resident lung microbiome. Lungs in patients with smoking history have undergone constant oxidative stress, resulting in irreversible lung damage. *M. avium*, like others in the genus, can adhere to damaged lung tissue, and additionally decrease the immune response of patients affected [43]. Corticosteroids are typically used as treatment for COPD and have proven success in alleviating inflammation. However, there are many indicators for a positive correlation between corticosteroid usage and increased rates of *M. avium* infections [44].

It is also crucial to understand that illnesses such as COPD cause detrimental imbalances that leave patients’ immune systems vulnerable to MAC infections. In COPD patients, specifically those with a history of smoking and exposure to inhaled toxins, there is high oxidative and carbonyl stress in the lungs. The oxidative burden surpasses the lung’s anti-oxidative defense, which causes chronic damage to the lung tissue [45]. This results in the production of reactive organic molecules that are responsible for accelerating the destruction of tissue, which then increases the carbonyl stress. The oxidative and carbonyl stress results in autoimmune disease and triggers the activation of pro-inflammatory markers. Excess oxidative stress is likely a factor contributing to a rise of MAC infections amongst COPD patients. Due to the oxidative stress, pulmonary tissue damage, anatomic changes, unregulated immune response to pathogens, dysregulation of cytokines, and increased inflammatory response in the body all occur, lowering the ability of the COPD patient population to contain mycobacterium infections [46]. Another imbalance that has significant repercussions is that of Glutathione [38]. Glutathione is an important thiol tripeptide antioxidant that maintains cellular redox state and cellular homeostasis in a variety of cellular processes within mammalian cells. An imbalance of Glutathione in COPD patients is detrimental because it damages cellular homeostasis and the metabolism of pulmonary tissues. The damage, done due to imbalances in the immune system, results in COPD patients having significantly higher susceptibility to MAC infections.

**Figure 1:**
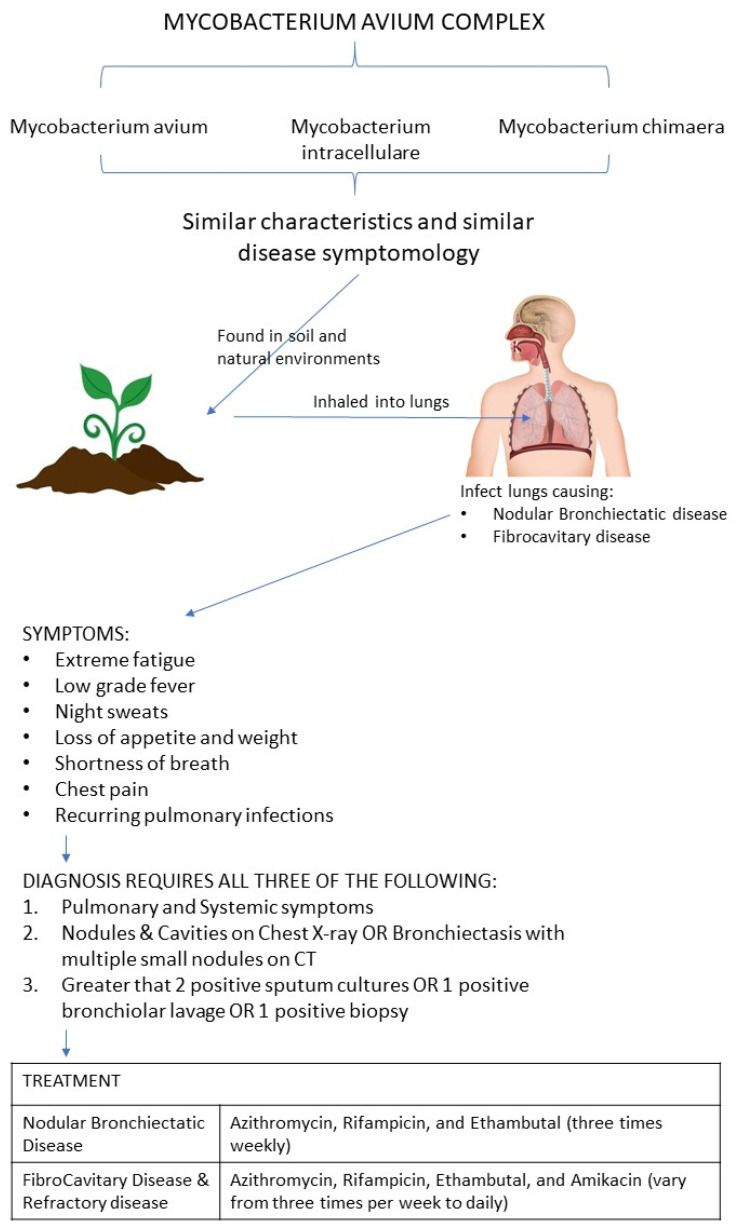
A figure elucidating the most common species making up MAC, MAC’s infectious route into the lungs, its symptoms, diagnosis, and treatment.

In this review, the diagnosis and treatment of MAC in the COPD patient population was examined in order to better understand their relationship [42]. Typically, MAC infections are diagnosed by sputum cultures and acid-fast staining. In initial phases, the treatment can consist of albuterol, ipratropium, prednisone, physiotherapy, and nutrition. However, antibiotic treatment can be used if conditions worsen (Refer to Figure 1). Patients are also advised to avoid exposure to soil and the environment since MAC is abundant there. The increased risk of opportunistic infections in COPD patients such as MAC makes it critical to consider diagnostic testing early in order to start the proper treatment. If treatment is delayed, the immune systems of COPD patients are left vulnerable to MAC colonization. Due to the increase in incidence of MAC infections and the high prevalence of COPD, it is important to consider aspects involved in risk factors, diagnosis, and potential contraindications in the treatment of those affected by both.

## Data Availability

Data sharing not applicable. No new data were created or analyzed in this study. Data sharing is not applicable to this article.
